# Hydrogel Materials for Drug Delivery and Tissue Engineering

**DOI:** 10.3390/polym17212862

**Published:** 2025-10-27

**Authors:** Aneta Ostróżka-Cieślik, Sławomir Wilczyński

**Affiliations:** 1 Department of Pharmaceutical Technology, Faculty of Pharmaceutical Sciences in Sosnowiec, Medical University of Silesia, Jedności Street 8b, 41-208 Sosnowiec, Poland; 2 Department of Basic Biomedical Science, Faculty of Pharmaceutical Sciences in Sosnowiec, Medical University of Silesia, Jedności Street 8b, 41-208 Sosnowiec, Poland; swilczynski@sum.edu.pl

## 1. Introduction

Hydrogels are a modern form of medicine consisting of water trapped in the structure of gel-forming polymers. They can mimic the biological properties of soft tissues and absorb significant amounts of body fluids while maintaining their spatial structure, making them ideal functional materials in dressing systems, drug carriers, and scaffolds used in tissue engineering. In recent years, there has been a dynamic development in polymer chemistry, which has led to the development of advanced hydrogel matrices with new functional properties. Stimuli-responsive hydrogels that react to a single stimulus (temperature, pH, ionic strength, redox reactions, light, or magnetic field) or several stimuli simultaneously and independently are the subject of numerous studies. This function makes them more susceptible to modification and controlled response after application in clinical conditions, where accelerated healing, regeneration of damaged tissues, and targeted cancer therapy are key [1,2]. Increasing attention is being paid to hydrogels developed on the basis of natural polymers such as chitosan, hyaluronic acid, collagen, alginates, pectin, and gelatin, which are non-toxic and characterized by biocompatibility, biodegradability, and a structure similar to the native extracellular matrix (ECM). Their unique properties make them particularly useful in tissue regeneration, drug delivery, wound healing, and scaffold production. Based on biopolymers, multifunctional composite hydrogels, hybrid hydrogels, and hydrogel microrobots, combining the properties of smart hydrogels and advanced microrobotics solutions has been developed [3,4].

Hydrogel biomaterials have been used in the formulation of bioinks for 3D/4D bioprinting. Thanks to the developed technological strategies, bio-gels show potential as matrices for the design of dressings, alternative drug delivery methods, and cell growth-promoting systems [3,5]. The use of Quality by Design (QbD) methods and in silico computer modeling tools enables the development of precise and more predictable hydrogel formulations. It is suggested that drug technology should take into account, among other things, the optimization of unit processes, critical formulation parameters, and controlling factors affecting quality at every stage of production [6,7].

Some of the innovations discussed were presented in the Special Issue of Polymers entitled ‘Hydrogel Materials for Drug Delivery and Tissue Engineering,’ which is introduced by this article.

## 2. Overview of Published Articles

Esparza-Villalpando et al. [8] developed an innovative drug delivery system (DDS) containing dexketoprofen (DXT) and chlorhexidine (CHX) for use in dental surgery to treat postoperative pain and prevent postoperative wound infections. The preparation consists of PLGA (poly(lactic-co-glycolic acid)) microspheres with incorporated chlorhexidine (MS CHX), which were embedded in a PEG (polyethylene glycol)-based hydrogel containing dexketoprofen (HG DXT). The formulations were combined in a 2:1 ratio (HG-DXT: MS-CHX). The authors conducted studies on the antibacterial activity of the preparation against *E. faecalis*, *E. coli*, *S. aureus*, and *C. albicans*, its biocompatibility, stability, and API release under in vitro conditions. The developed system showed antimicrobial efficacy, optimal biocompatibility (cell viability was 80%), and stability during 50 days of storage. In the pharmaceutical availability study, dexketoprofen was released from the carrier quickly (rapid analgesic effect), while chlorhexidine was released in a prolonged manner (long-lasting antimicrobial effect). The authors suggest that the developed antiseptic-analgesic DDS shows potential for use in clinical practice.

In the second study [9], the authors developed a dermatological hydrogel insulin carrier based on Sepineo™ P 600 and Sepineo™ PHD 100 for the treatment of chronic wounds. The preformulation studies of the developed preparations confirmed that insulin is released in a sustained manner. After 6.5 h, 53.36% and 47.4% of the hormone were released from the Sepineo™ P 600 and Seppineo™ PHD 100-based hydrogel through the Strat-M^®^ membrane, respectively. Rheological analysis showed that the tested hydrogels belong to non-Newtonian, shear-thinning systems with a yield stress. The results obtained may serve as a starting point for further preclinical and clinical studies. An innovative dressing for use in chronic wound therapy was also proposed by da Silva et al. [10]. The authors combined a bacterial cellulose-based dressing with melatonin. The study was conducted in a rat model with streptozotocin-induced diabetes. The animals were divided into four groups: a control group, a group treated with a commercial bacterial cellulose-based dressing, and a group treated with a bacterial cellulose-based dressing with melatonin. The therapy was conducted over 14 days, and its effectiveness was assessed based on the analysis of pro-inflammatory cytokine concentrations (interleukin-6/IL-6, tumor necrosis factor-alpha/TNF-α, vascular endothelial growth factor/VEGF), PCNA (proliferating cell nuclear antigen), and types I and III collagen. In the group of rodents treated with a bacterial cellulose dressing with melatonin, reduced levels of IL-6, TNF-α, and VEGF, increased expression of PCNA; and an increase in type I collagen concentration. The proposed system has anti-inflammatory properties, promotes cell proliferation, and stimulates collagen production.

In the next article, Sun et al. [11] discussed the potential of hydrogels in inflammatory bowel diseases, with particular emphasis on ulcerative colitis and Crohn’s disease. The authors discussed the therapeutic potential of hydrogels (in the form of injectable hydrogels, hydrogel composites, and microforms incorporated into a hydrogel matrix) in three aspects: API delivery (oral, rectal, and parenteral), anti-inflammatory action, and intestinal mucosal regeneration. They suggest that hydrogels are an effective carrier of biomolecules that support intestinal epithelial regeneration. However, further research is needed to optimize formulations, ensure API stability in the matrix, and reduce the cost of manufacturing hydrogel preparations.

Mohseni-Motlagh et al. [12] suggest that the application of the Quality by Design (QbD) concept is one of the potential solutions to the technological problems of hydrogel preparation. The use of QbD allows for product quality control at every stage of manufacturing. The key elements of the QbD concept are defining the Quality Target Product Profile (QTPP), selecting Critical Quality Attributes (CQA), identifying Critical Material Attributes (CMA) and Critical Process Parameters (CPP), risk assessment and Design Of Experiments (DoE), and production control strategies. The authors reviewed studies on hydrogels that used the Quality by Design strategy. They suggest that the implementation of the QbD methodology promotes the optimization of production processes and the quality of manufactured preparations.

Further studies confirm the effectiveness of hydrogels in tissue reconstruction and regeneration. Nogoceke et al. [13] evaluated the effect of a commercial peptide hydrogel (Puramatrix™) on the behavior and differentiation capacity of hASC (human adipose-derived stem cells) cartilage cells. The authors confirmed that the preparation is characterized by a porous matrix, which promotes the adhesion and proliferation of hASC in vitro. During culture on the hydrogel, hASC cells showed the ability to spontaneously form cell aggregates. The Puramatrix™ hydrogel can stimulate chondrogenic gene expression and induce GAG (glycosaminoglycan) production. The authors suggest that the hydrogel has the potential to form functional cartilage tissue structures. Mohd Razak’s team [14] undertook innovative research into intervertebral disk (IVD) regeneration in patients with osteoarthritis. The authors investigated the therapeutic potential of a hydrogel based on hyaluronic acid (HA) and type II collagen (COLII). The study was conducted in a rat model with surgically induced intervertebral disk injury in the caudal region. The rodents were divided into three groups: a control group, a group with injury without treatment, and a group with injury that was treated with HA/COLII hydrogel (4 µL of hydrogel; HA/COLII concentration was 2 mg/mL). It was found that hydrogel implantation reduced abnormal nerve fiber growth after injury, improved hydration in the disks, and reduced pain sensitivity in the von Frey test. The authors conclude that hydrogel promotes intervertebral disk regeneration.

In a review article by Jeon et al. [15], the potential of adhesive hydrogels in the treatment of vascular diseases, bleeding control, and closure of postoperative wounds was highlighted. The limitations in the formulation of hydrogel matrices in terms of mechanical strength, biocompatibility, and API delivery to the site of action were analyzed. Innovative solutions in hydrogel technology and their impact on the effectiveness of vascular and wound therapies were discussed. The authors suggest the need to continue the scientific work included in the review in clinical trials.

## 3. Summary and Future Outlook

This Special Issue focuses on advances in the development of innovative hydrogels as drug carriers supporting wound therapy and tissue regeneration. It has been suggested that smart hydrogels, whose preparation technology is based on polymers sensitive to environmental stimuli, may revolutionize therapeutic interventions. Future research should focus on transferring results from preclinical stages to clinical trials and on standardizing QbD methodologies.
